# Metabolic Dysfunction‐Associated Steatotic Liver Disease (MASLD): Mechanisms, Clinical Implications and Therapeutic Advances

**DOI:** 10.1002/edm2.70132

**Published:** 2025-11-19

**Authors:** Dalia M. Miller, Kiana F. McCauley, Kimberly J. Dunham‐Snary

**Affiliations:** ^1^ Department of Medicine Queen's University Kingston Ontario Canada; ^2^ Bachelor of Health Sciences Program Queen's University Kingston Ontario Canada; ^3^ Department of Biomedical & Molecular Science Queen's University Kingston Ontario Canada

**Keywords:** mitochondrial dysfunction‐associated steatohepatitis (MASH), mitochondrial dysfunction‐associated steatotic liver disease (MASLD), mitochondrial genetics, Western‐style diet

## Abstract

**Introduction:**

Metabolic Dysfunction‐Associated Steatotic Liver Disease (MASLD) has emerged as the most prevalent chronic liver disease worldwide, affecting ~25%–30% of the adult population, with higher prevalence observed in individuals with obesity and type 2 diabetes. Among reported MASLD cases, prevalence is consistently higher in men than in women, and global incidence has risen by ~50% over the past two decades, mirroring the global rise in obesity and metabolic syndrome. MASLD encompasses a spectrum of hepatic pathologies ranging from simple steatosis to steatohepatitis, fibrosis and cirrhosis. Despite its high prevalence, the heterogeneity in disease progression and relative absence of approved pharmacological therapies pose challenges for effective clinical management.

**Methods and Results:**

This review synthesises current literature on MASLD across epidemiology, pathophysiology, clinical presentation and treatment. Key molecular mechanisms, including lipid metabolism dysregulation, insulin resistance and mitochondrial dysfunction, are examined with a focus on understanding the basis for progression to metabolic dysfunction‐associated steatohepatitis (MASH). Clinical manifestations, diagnostic tools and risk stratification systems for MASLD are summarised. Current and emerging therapies such as lifestyle interventions, pharmacological agents and microbiome‐targeted strategies are reviewed. The review also highlights ongoing challenges, including diagnostic limitations, disease heterogeneity and disparities in care.

**Conclusion:**

MASLD is a complex, multifactorial liver disease with a growing public health impact, driven by the rising prevalence of metabolic syndrome. Mitochondrial dysfunction is a critical nexus linking genetic susceptibility to metabolic stress and inflammatory responses. Preclinical models that capture these mitochondrial contributions are vital for therapeutic discovery and for advancing personalised medicine approaches in MASLD care.

## Graphical abstract

Healthy liver function depends on the coordinated activity of hepatocytes, Kupffer cells, stellate cells, endothelial cells and cholangiocytes. During MASLD, all cell types undergo pathological changes, driving steatosis, inflammation, fibrosis, impaired exchange and ductular reactions. This work highlights how diverse liver cells cooperatively fuel disease progression and compromise organ function.

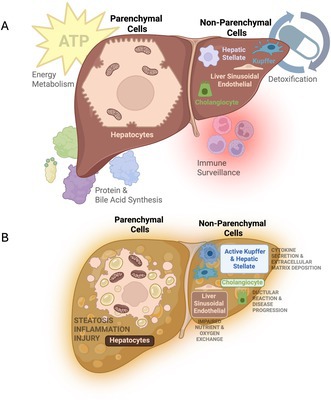

## 1 Introduction

**FIGURE 1:**
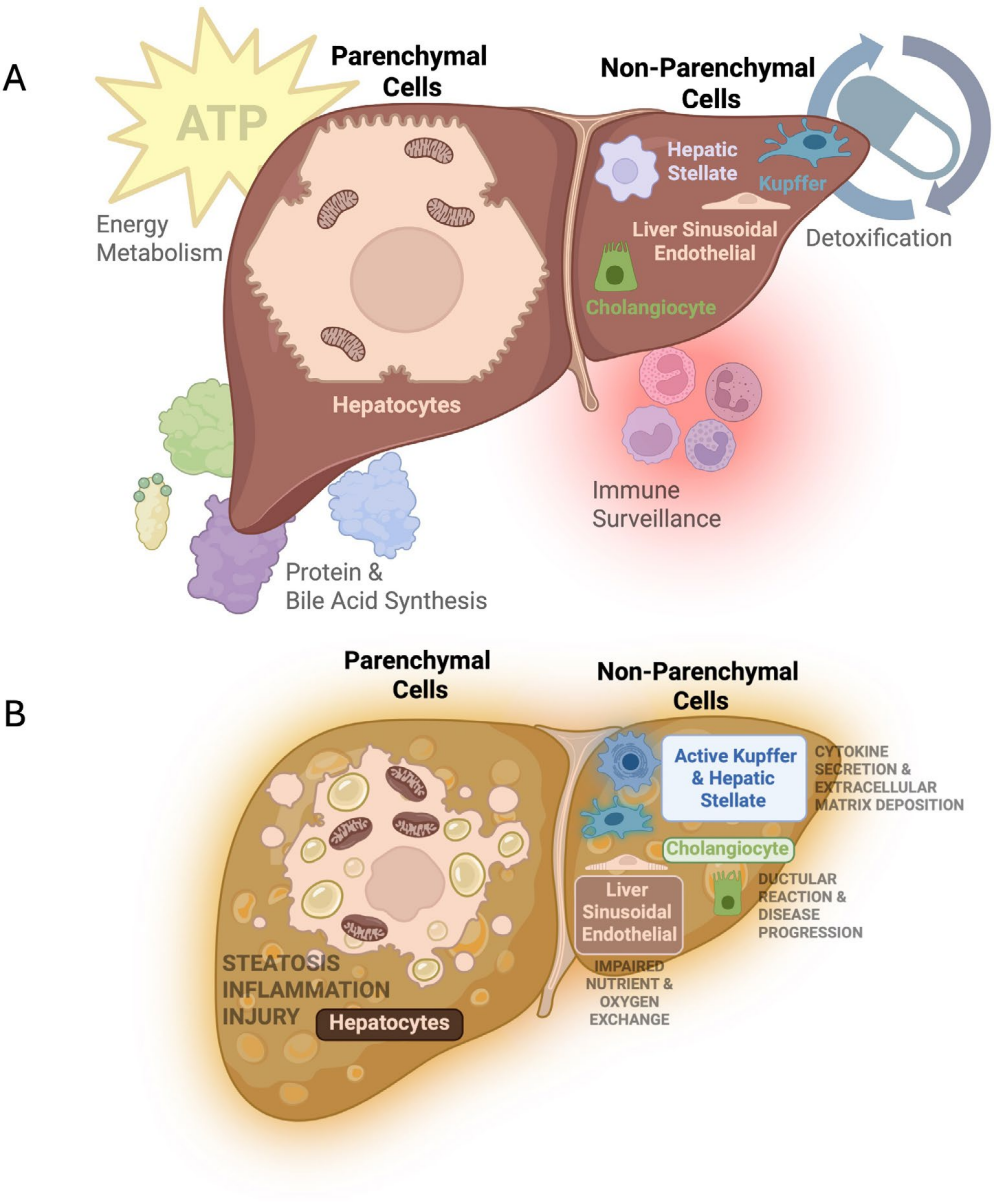
Diverse liver cell types cooperatively drive health and contribute to MASLD pathogenesis. (A) In healthy liver tissue, distinct parenchymal (hepatocytes) and non‐parenchymal (Kupffer, hepatic stellate, liver sinusoidal endothelial and cholangiocytes) cell types coordinate essential functions, including energy metabolism, detoxification, immune surveillance and protein and bile acid synthesis. (B) During MASLD progression, all major liver cell types undergo pathological changes. Hepatocytes accumulate fat and become inflamed and injured. Kupffer cells and hepatic stellate cells are activated, promoting inflammation and fibrosis through cytokine secretion and extracellular matrix deposition. Liver sinusoidal endothelial cells lose their fenestrations (capillarisation) and develop basement membranes, impairing nutrient and oxygen exchange. Cholangiocytes expand and remodel, contributing to ductular reactions and disease progression.

A critical factor in metabolic dysfunction‐associated steatotic liver disease (MASLD) pathophysiology is mitochondrial dysfunction, or mitochondriopathy, which impairs lipid metabolism leading to lipid accumulation, and increases in oxidative stress, reducing energy production [1]. These processes occur within the highly specialised microenvironment of the liver, a multifunctional organ responsible for regulating systemic energy metabolism, detoxification, immune surveillance and synthesis of essential proteins and bile acids [2]. Central to this role is the liver's regulation of glucose homeostasis, achieved through glycogen storage and mobilisation, gluconeogenesis and integration of fatty acid metabolism, positioning it as a key determinant of whole‐body energy balance [3]. The interplay between diverse cell types within the liver is critical in the progression from simple steatosis to inflammation and fibrosis (Figure 1). Hepatocytes, the primary parenchymal cells, constitute approximately 70%–80% of the liver's mass and play a central role in lipid and glucose homeostasis [4]. Non‐parenchymal cells, including Kupffer cells (resident macrophages), hepatic stellate cells (HSCs), liver sinusoidal endothelial cells (LSECs) and cholangiocytes, support immune regulation, fibrogenesis, vascular integrity and bile secretion, respectively [5, 6].

**FIGURE 2:**
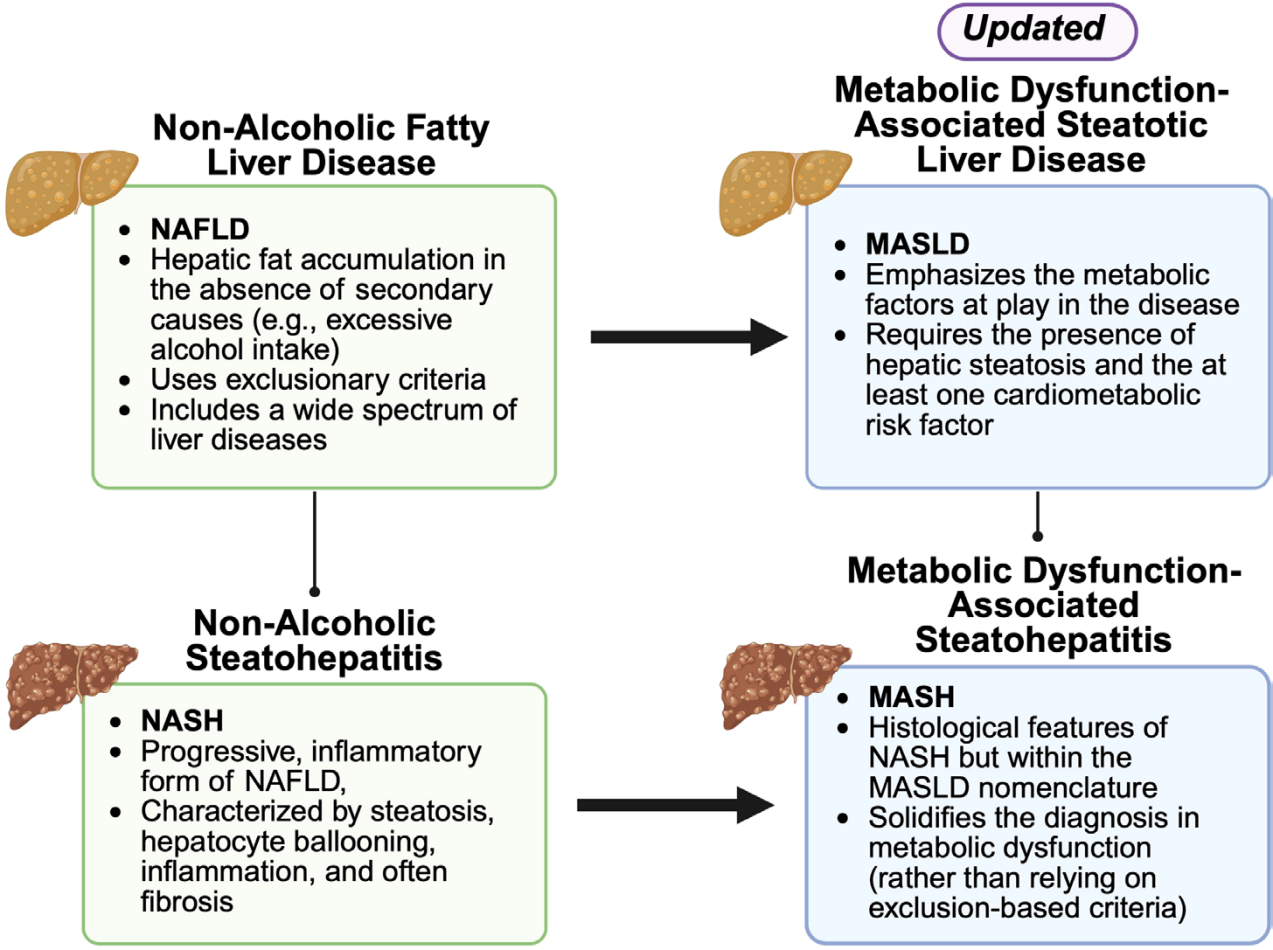
Transition from NAFLD/NASH to MASLD/MASH nomenclature. Updated terminology for fatty liver disease, reflecting the shift from exclusion‐based definitions (NAFLD and NASH) to a framework grounded in metabolic dysfunction (MASLD and MASH).

MASLD, formerly known as non‐alcoholic fatty liver disease (NAFLD), represents a spectrum of liver pathologies characterised by excessive fat accumulation in hepatocytes in the absence of significant alcohol consumption, and is associated with metabolic syndrome (defined as the presence of at least three of: elevated waist circumference, impaired fasting glucose, hypertension, hypertriglyceridemia or low HDL cholesterol) [7] (Figure 2). The recent shift in nomenclature from NAFLD to MASLD reflects a more accurate framing of the condition as a metabolic disorder, centering diagnostic criteria on metabolic dysfunction rather than exclusion of alcohol use [8]. This reclassification aims to improve diagnostic clarity and align with emerging evidence linking liver disease to systemic metabolic derangements. A subset of MASLD patients develops metabolic dysfunction‐associated steatohepatitis (MASH), formerly known as non‐alcoholic steatohepatitis (NASH), a more severe form of the disease marked by hepatic inflammation and fibrosis, which significantly increases the risk of progression to cirrhosis or hepatocellular carcinoma (HCC) [9].

Both genetic and metabolic factors impact MASLD progression and severity. For example, cardiometabolic diseases, which comprise a constellation of obesity, insulin resistance, dyslipidemia and hypertension, are well‐established drivers of MASLD progression [10, 11]. Insulin resistance promotes hepatic lipogenesis (excessive fatty acid synthesis) and impairs lipid clearance, while dyslipidemia exacerbates hepatic fat deposition [12, 13]. Systemic hypertension also further contributes to disease progression through vascular inflammatory mechanisms [14].

### 1.1 Lifestyle Factors

Beyond genetic and metabolic risk factors, lifestyle and environmental factors such as high‐sugar and high‐fat diets, food insecurity, physical inactivity, poor sleep hygiene, circadian rhythm disruption and chronic stress can accelerate MASLD onset and severity. These risk factors highlight the importance of early intervention strategies targeting both metabolic and lifestyle modifications. Their combined effects exacerbate systemic inflammation, oxidative stress and insulin resistance, creating a pro‐steatotic and pro‐fibrotic hepatic environment [15, 16, 17].

A ‘Western‐style’ diet, characterised by high fat (~30% of total caloric intake) and high sugar (~10% of total calories, largely from added sucrose or high fructose corn syrup), is a known risk factor for cardiometabolic disease and accelerates MASH development in both preclinical and clinical settings [18]. This dietary pattern promotes metabolic dysregulation by inducing insulin resistance, chronic inflammation and oxidative stress, all central to cardiometabolic disease pathogenesis [19, 20, 21]. It also accelerates hepatic lipid accumulation, mitochondrial dysfunction and fibrogenesis, hallmarks of MASH [19, 20]. In 2023, Yang et al. emphasised how a Western‐style diet interacts with gut microbiota to promote MASLD development. A choline‐low, high‐fat, high‐sugar diet effectively induces MASH in mice, replicating disease features by remodelling the gut microbiota, indicating *Blautia producta* and the metabolite 2‐oleoylglycerol as pivotal diet‐related contributors to MASH [22]. These mediators drive liver inflammation and fibrosis through the GPR119/TAK1/NF‐κB/TGF‐β1 signalling pathway, which primes macrophages and activates hepatic stellate cells [22].

Physical inactivity is strongly associated with MASLD and its progression to MASH, independent of weight status [23]. Sedentary behaviour reduces mitochondrial biogenesis, increases insulin resistance and contributes to lipotoxicity [23]. Conversely, structured physical activity (e.g., moderate‐to‐vigorous aerobic exercise for 135–240 min/week) improves hepatic steatosis and reduces intrahepatic triglycerides, even without significant weight loss [24]. Emerging evidence also suggests that sleep quality and circadian rhythm disruption play a significant role. A meta‐analysis of 261,544 participants showed that short sleep duration (< 5–6 h) correlates with increased MASLD risk, insulin resistance and hepatic steatosis [25]. Animal studies using circadian‐disrupted models (e.g., *Clock* or *Bmal1* knockout mice) demonstrate that circadian misalignment (commonly seen in shift workers, those experiencing chronic jet lag or individuals with sleep fragmentation) exacerbates hepatic lipid accumulation and bile acid dysregulation [26]. Supporting this, a Korean study of 5661 participants found that long work hours (53–83 h/week) increased MASLD odds by 23% versus standard hours (36–42 h/week), independent of confounders (e.g., BMI, smoking, alcohol use and exercise) [27]. Moreover, chronic psychological stress, via hypothalamic–pituitary–adrenal axis activation and glucocorticoid release, also promotes visceral adiposity, glucose dysregulation and hepatic triglyceride synthesis [28, 29].

Together, these findings highlight how MASLD is influenced not only by intrinsic metabolic factors, but also by a complex interplay of behavioural and environmental exposures. The convergence of poor diet, inactivity, sleep disruptions, circadian misalignment and stress accelerates liver injury, emphasising the need for holistic and multifactorial approaches to both MASLD prevention and treatment.

### 1.2 Global Epidemiology and Prevalence

MASLD now affects nearly 1.3 billion adults worldwide, with prevalence estimates of 25%–30% in the general population and up to 70% among people with type 2 diabetes [30, 31]. Age‐specific analyses reveal that MASLD prevalence increases from 7% to 14% in children to ~38% in adults, reflecting both cumulative metabolic insults and age‐related declines in mitochondrial and immune function [32]. Alarmingly, paediatric MASLD is on the rise, with an estimated global prevalence increasing from 4.62% in 2000 to 8.77% in 2021, and reaching 52.5% among obese children, underscoring the need for early‐life interventions [33].

**FIGURE 3:**
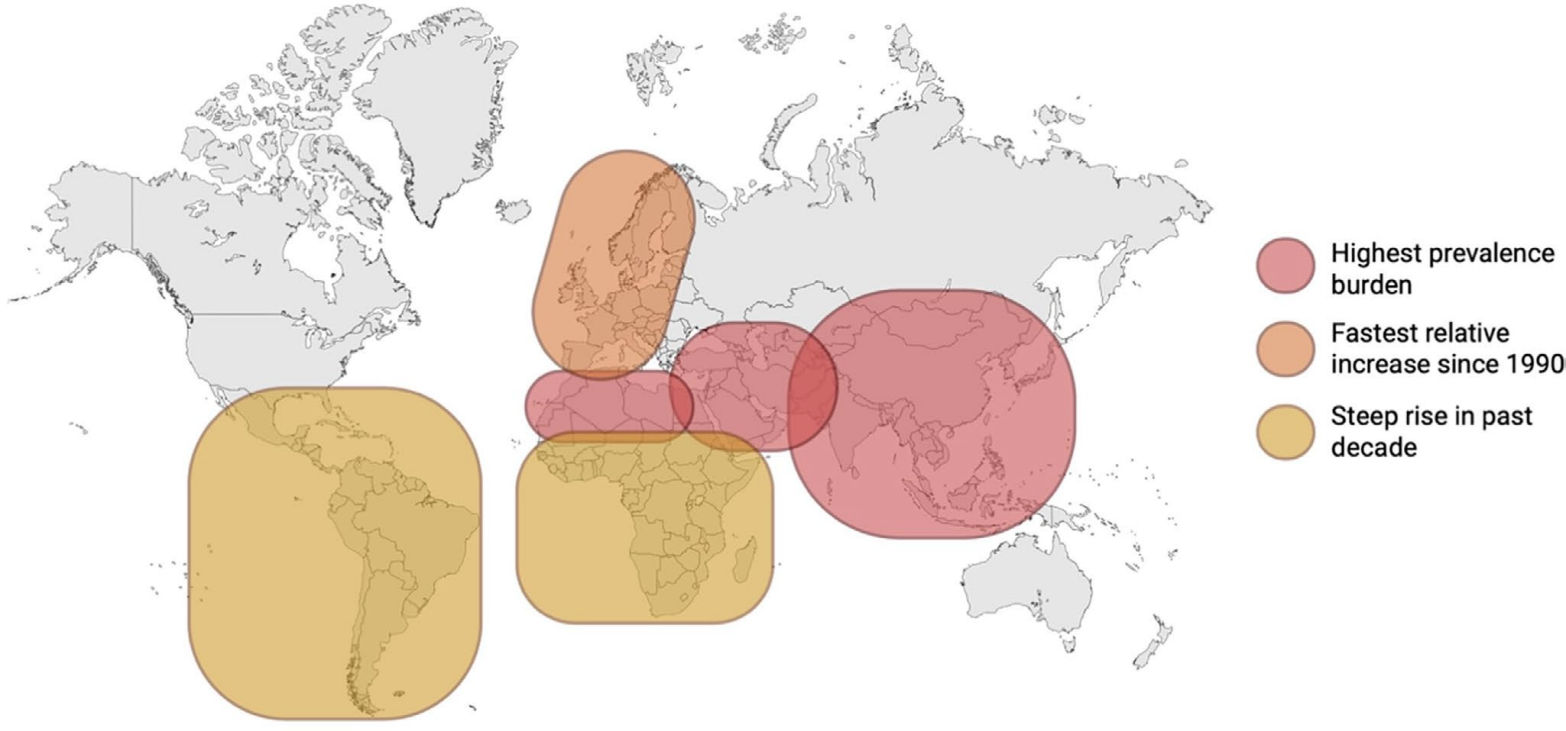
Global distribution of MASLD cases, burden and trends in prevalence. Geographic patterns in metabolic dysfunction‐associated steatotic liver disease (MASLD) epidemic. Countries in red represent regions with the highest current prevalence burden, notably in East Asia, South Asia and Middle East/North Africa. Regions in orange, including much of Western Europe, demonstrate the fastest relative increase in MASLD cases since 1990. Areas in yellow, primarily in sub‐Saharan Africa and Latin America have experienced a steep rise in recent years, driven by urbanisation, dietary shifts and decreased physical activity.

Regionally, the highest prevalence burdens are observed in East Asia, South Asia and the Middle East/North Africa, while Western Europe is experiencing the most rapid relative increase in case numbers since 1990 [31]. Additionally, low‐ and middle‐income countries, including those in sub‐Saharan Africa and Latin America, have witnessed steep rises in MASLD over the past decade, driven by rapid urbanisation, dietary shifts towards high‐caloric foods and reduced physical activity [34]. Disease modelling projects that by 2040, more than 55% of adults worldwide will be affected by MASLD, and 20%–30% will progress to MASH [35] (Figure 3).

MASLD carries a substantial healthcare burden worldwide. In the United States, per‐patient direct costs average $1613 USD; in Europe, the range is €354–€1163 across Germany, France, Italy and the United Kingdom, largely due to hospitalizations, imaging and management of comorbidities [36]. In Canada, national expenditures reached $3.76 billion CAD in 2020, and are projected to nearly double by 2050 [37] Globally, direct healthcare costs associated with MASLD and its complications exceed $100 billion USD annually, with indirect costs, such as lost productivity and disability, substantially adding to this [37]. Comorbid cardiometabolic diseases amplify both MASLD prevalence and severity. The majority of MASLD patients exhibit features of metabolic dysfunction, and MASLD doubles the risk of cardiovascular events and chronic kidney disease [30, 38]. Moreover, MASH, the progressive form of MASLD, is recognised as one of the leading indications for liver transplantation globally, accounting for ~20.3% of all transplants in Western countries, rivalling hepatitis C and alcohol‐related liver disease [35, 39]. This shifting landscape underscores the urgent need for public health strategies that address MASLD as a global public health problem.

### 1.3 Clinical Importance of Early Detection

**FIGURE 4:**
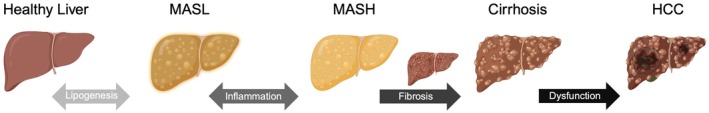
Progression of metabolic‐dysfunction steatotic liver disease (MASLD). Natural progression of MASLD, beginning with a benign healthy liver and progressing through the reversible stages of lipogenesis resulting in metabolic dysfunction‐associated steatotic liver (MASL), and inflammation leading to metabolic dysfunction‐associated steatohepatitis (MASH). Then the beginning of the irreversible stages, starting with fibrosis, cirrhosis and ultimately hepatocellular carcinoma (HCC). This continuum highlights the escalating severity of liver damage driven by metabolic dysfunction and underscores the urgency of early detection and intervention.

Timely diagnosis of MASLD and effective intervention are critical to preventing its progression to more severe conditions, including fibrosis, cirrhosis and HCC (Figure 4). Many individuals with MASLD remain asymptomatic until advanced stages, highlighting the need for improved screening protocols and non‐invasive biomarkers [40]. However, diagnosis alone is not sufficient, as without effective, accessible interventions, early detection offers little clinical value. Early detection allows for targeted interventions, including lifestyle modifications and pharmacological approaches, to mitigate disease progression [41]. Identifying high‐risk individuals enables proactive management strategies to reduce long‐term hepatic and cardiovascular complications.

Diagnosing MASLD depends on identifying hepatic steatosis through imaging alongside at least one cardiometabolic risk factor, while ruling out other liver diseases or secondary causes such as heavy alcohol use [42, 43]. In contrast, diagnosing MASH, the more advanced form of MASLD, requires signs of hepatocellular damage such as lobular inflammation and hepatocyte ballooning [42, 43]. Despite its diagnostic utility in MASH, liver biopsy is rarely performed in clinical practice unless the disease is suspected to be advanced or there is diagnostic uncertainty, due to its invasiveness, cost and sampling variability. These limitations underscore the urgent demand for accurate, non‐invasive diagnostic tools [43]. One such surrogate is vibration‐controlled transient elastography (FibroScan), which is increasingly used to assess liver stiffness (which estimates histological fibrosis levels) and detect early‐stage fibrosis or cirrhosis non‐invasively. Beyond its risk of progression to irreversible conditions such as cirrhosis, HCC and liver failure, MASH further disrupts metabolic homeostasis, contributing to comorbidities like obesity and diabetes through chronic inflammation and fibrosis. This self‐perpetuating cycle of worsening disease underscores the importance of developing early detection methods and targeted therapies [43].

## 2 Clinical Presentation

### 2.1 Spectrum of MASLD

MASLD represents a spectrum of liver conditions driven by metabolic dysfunction, ranging from simple steatosis to more advanced disease in which there is also inflammation and fibrosis. MASLD is a prevalent chronic liver disorder, often asymptomatic in its early stages and is defined by the accumulation of fat in at least 5% to 10% of hepatocytes without other identifiable liver diseases [44].

Simple steatosis, is characterised by the accumulation of triglycerides within hepatocytes without inflammation, and is often asymptomatic being detected incidentally through imaging [42, 45]. However, some patients with steatosis experience symptoms, including fatigue, upper abdominal discomfort, bloating and nonspecific gastrointestinal complaints [44]. While traditionally considered benign, longitudinal studies indicate that even simple steatosis may progress to more severe disease under conditions such as metabolic syndrome, insulin resistance or genetic predisposition. Importantly, non‐metabolic factors, including long‐term use of medications such as corticosteroids (e.g., prednisone), tamoxifen or methotrexate, can also contribute to hepatic fat accumulation, underscoring the diverse etiologies of steatosis beyond metabolic dysfunction [46]. Additionally, systemic manifestations of MASLD, such as daytime sleepiness, cognitive impairment and autonomic nervous system dysfunction, are increasingly recognised and can significantly impair quality of life, independent of liver disease severity. Cognitive changes are common in this population, with up to half of patients reporting mild cognitive symptoms, and among those affected, 46% experience impairments that are moderate to severe. Moreover, 43% report a history of falls, with significantly higher rates of recurrent falls, injuries, emergency visits, fractures and hospitalizations compared to matched controls [47]. These symptoms suggest broader neurological and functional consequences of MASLD and highlight the need for early recognition and targeted intervention to reduce morbidity and improve daily functioning.

MASH is a more advanced form of MASLD, characterised by hepatocellular injury, inflammation, ballooning degeneration and metabolic dysfunction [42]. A feature of MASH is elevated liver enzymes, such as ALT (alanine transaminase) and AST (aspartate transaminase) [42, 45]. Histological features include hepatocyte ballooning, lobular inflammation and varying degrees of fibrosis, which may ultimately lead to cirrhosis or HCC. MASH is often associated with systemic metabolic dysfunction, including obesity, type 2 diabetes and dyslipidemia. Together, these stages underscore the heterogeneous nature of MASLD and highlight the importance of early recognition, risk stratification and tailored clinical management to prevent disease progression and long‐term complications (Table 1).

**TABLE 1:** Clinical, histological and laboratory differences between simple steatosis and MASH.

Feature	Simple steatosis	MASH
Clinical presentation	Often asymptomatic; may present with fatigue, bloating, mild upper abdominal discomfort or nonspecific GI complaints; associated with metabolic syndrome	Symptoms may overlap with steatosis but can include more pronounced fatigue; often associated with obesity, type 2 diabetes and dyslipidaemia; increased risk of cirrhosis‐related complications
Histological findings	≥ 5%–10% hepatocyte fat accumulation without inflammation or fibrosis	Steatosis plus hepatocyte ballooning, lobular inflammation and variable fibrosis; may progress to cirrhosis or HCC
Laboratory findings	Mild elevation in ALT/AST; may be detected incidentally	Elevated ALT/AST, possible rise in inflammatory markers; abnormalities in glucose and lipid profiles
Prognosis	Traditionally considered benign, but may progress, especially with metabolic risk factors	Higher risk of progression to cirrhosis (~15%–25% within 8–13 years), HCC (~7% of cirrhosis patients within 1‐year), transplantation or mortality (50% of HCC patients)

*Note:* Summary of key distinguishing features between simple steatosis and metabolic dysfunction‐associated steatohepatitis (MASH), the two primary stages within the MASLD spectrum. ALT/AST ratio reflects liver health, with a ratio > 1 indicating MASLD, while a ratio > 2 indicating alcoholic liver disease and < 1 indicating other liver issues or less severe damage. Differences are highlighted across clinical presentation, histological characteristics, laboratory findings and prognostic implications to aid in diagnostic and therapeutic decision‐making.

### 2.2 Progression to Advanced Liver Disease

Disease progression in MASLD is heterogeneous, with longitudinal data suggesting that 12%–40% of individuals with metabolic dysfunction‐associated steatotic liver (MASL) will progress to MASH within 8–13 years. Among those with MASH and fibrosis, ~15%–25% progress to cirrhosis within the same timeframe. Further, ~7% of patients with compensated cirrhosis due to MASLD will develop HCC within 10 years, and 50% of these individuals will require liver transplantation or experience liver‐related mortality [48]. Understanding fibrosis staging is essential for risk assessment and long‐term management [49]. The progression of MASLD varies among individuals, with advanced fibrosis being the strongest predictor of liver‐related morbidity and mortality. While some patients remain stable, others may develop end‐stage liver disease or require liver transplantation, with an estimated 3%–5% of MASL cases advancing to cirrhosis [50, 51]. Unlike other chronic liver diseases, MASLD is primarily driven by metabolic dysfunction rather than viral or autoimmune processes. Despite its slow progression, the increasing global prevalence of obesity and diabetes highlights its significance as a primary public health concern [49].

MASLD manifestation ranges from benign hepatic steatosis to advanced liver disease. Fibrosis staging is a key determinant of disease progression, with patients who develop bridging fibrosis or cirrhosis at the highest risk for liver‐related morbidity and mortality [49]. Advanced fibrosis is the strongest predictor of poor outcomes, and some patients may progress to cirrhosis, end‐stage liver disease or HCC [51]. While most individuals with MASLD do not develop significant liver complications, identifying those at greatest risk is critical. Current best practices involve a stepwise, non‐invasive evaluation beginning with a blood surrogate score (e.g., FIB‐4), If patients are intermediate or high risk, this is followed by imaging‐based liver stiffness assessments using ultrasound elastography [52]. MASLD is also associated with several extrahepatic complications, most notably an increased risk of cardiovascular disease, which remains the leading cause of mortality in affected individuals, driven by systemic inflammation, dyslipidemia and insulin resistance [53]. Additionally, those with MASLD have a higher prevalence of chronic kidney disease with shared risk factors such as hypertension and diabetes, and polycystic ovarian syndrome likely due to underlying insulin resistance and metabolic dysfunction [54]. These systemic effects highlight the need for comprehensive management strategies that address hepatic and extrahepatic manifestations of the disease.

Research has shown that patients with advanced fibrosis or compensated cirrhosis face a substantial likelihood of disease progression within a short timeframe, with an estimated 20% developing cirrhosis or decompensation over 2 years [52]. The presence of portal hypertension, increased collagen deposition and hepatic stellate cell activation are markers of heightened fibrogenesis, contributing to a greater risk of disease advancement [52]. While tools like the Child‐Pugh and MELD (Model for End‐Stage Liver Disease) scores are widely used to assess prognosis in cirrhosis, their utility is limited to later stages and not applicable for earlier phases of MASLD. Current non‐invasive serum biomarkers have only moderate accuracy in predicting disease progression, necessitating continued research to improve risk stratification methods [52].

Given the growing global prevalence of obesity and diabetes, MASLD represents a significant public health challenge. Early identification of high‐risk individuals, combined with targeted lifestyle interventions and emerging pharmacological therapies, is essential for preventing progression to severe liver disease and reducing overall morbidity [52].

## 3 Pathogenesis

MASLD is a complex metabolic disorder driven by multiple interrelated pathophysiological processes, including lipid metabolism [55], insulin resistance [56], mitochondrial dysfunction [57], gut‐liver axis alterations [58] and chronic inflammation [59]. These mechanisms contribute to hepatic steatosis, inflammation and fibrosis, ultimately leading to advanced liver disease in a subset of patients.

### 3.1 Lipid Metabolism Dysregulation

**FIGURE 5:**
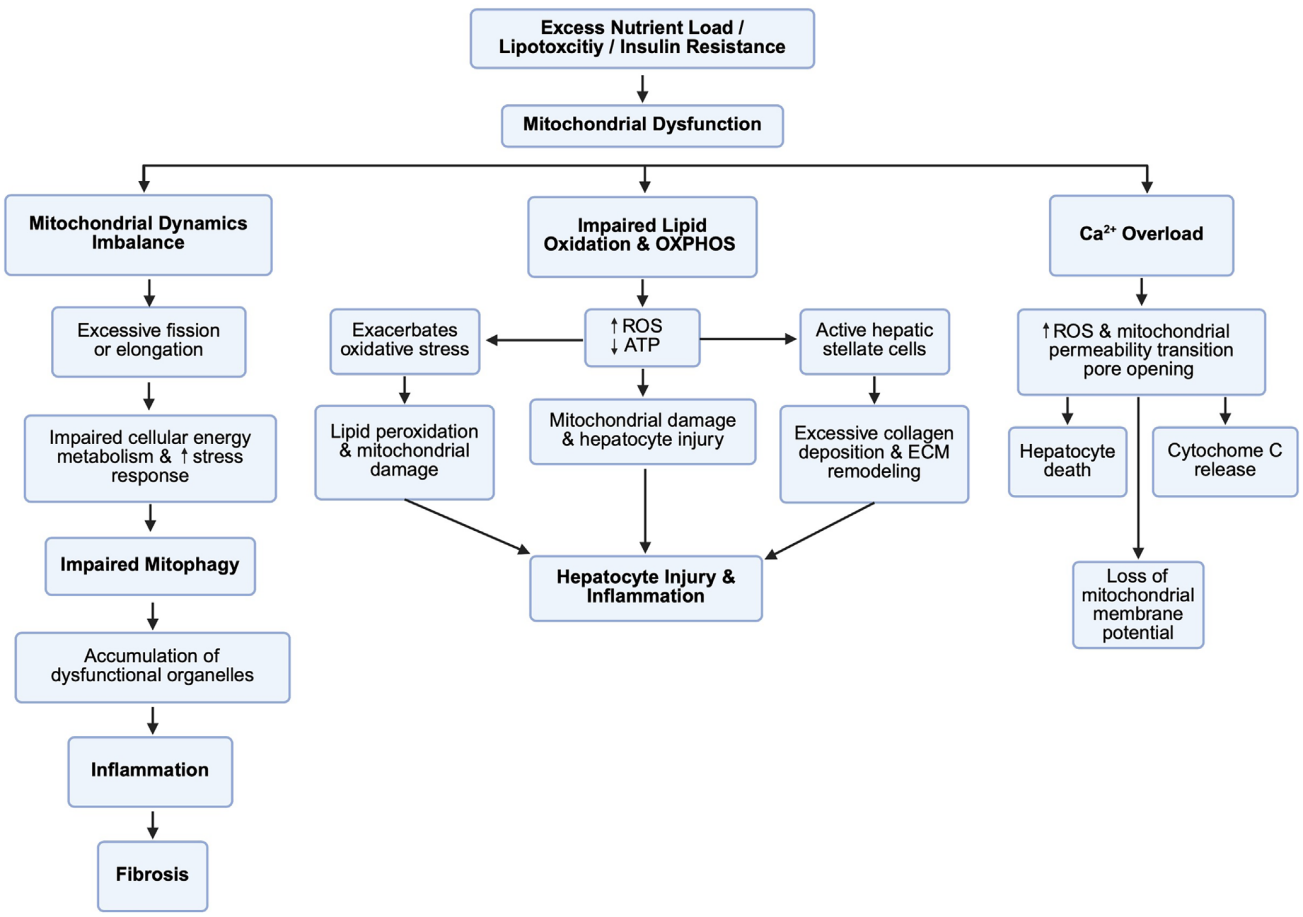
The interplay of mitochondrial fragmentation, impaired mitophagy, oxidative stress and apoptosis in MASLD. The interconnected pathways contributing to mitochondrial dysfunction in Metabolic Dysfunction‐Associated Steatotic Liver Disease (MASLD). Disrupted mitochondrial dynamics, particularly excessive fragmentation and impaired fusion, impair energy metabolism and promote oxidative stress. Impaired mitophagy leads to the accumulation of dysfunctional mitochondria, further exacerbating inflammation and fibrosis. Impaired lipid oxidation and oxidative phosphorylation (OXPHOS) increase reactive oxygen species (ROS) and decrease energy production. These changes promote oxidative stress, mitochondrial damage and hepatocyte injury, while also activating hepatic stellate cells, which contribute to fibrogenesis and amplify hepatic inflammation. Mitochondrial calcium (Ca^2+^) overload triggers additional ROS production and opening of the mitochondrial permeability transition pore, resulting in hepatocyte death, loss of membrane potential and cytochrome c release.

One of the primary features of MASLD is the disruption of lipid homeostasis, characterised by excessive hepatic lipid accumulation due to increased de novo lipogenesis and impaired lipid clearance [60]. De novo lipogenesis is the process by which the liver synthesises fatty acids from carbohydrates and is upregulated in MASLD, particularly in the setting of insulin resistance and excessive carbohydrate intake [61, 62, 63]. Hyperinsulinemia, (excess insulin in the blood) and elevated glucose levels activate sterol regulatory element‐binding protein 1 c (SREBP‐1c) and carbohydrate‐response element‐binding protein (ChREBP), key transcription factors that drive lipid synthesis [64, 65]. SREBP‐1c is activated by excessive insulin levels and is a transcription factor that regulates genes involved in fatty acid synthesis and glucose metabolism [64], while ChREBP is activated by glucose and is a transcription factor that plays a crucial role in regulating glucose and lipid metabolism [65]. Concurrently, impaired adipose tissue lipolysis results in increased free fatty acid flux to the liver, further exacerbating hepatic steatosis [66]. The liver's inability to efficiently export triglycerides as very‐low‐density lipoproteins further contributes to lipid accumulation [67]. These disruptions in lipid synthesis, transport and clearance form the metabolic foundation of hepatic steatosis in MASLD and consequently set the stage for subsequent mechanisms of liver injury and dysfunction, including fibrosis. Taken together, the dysregulation of lipid metabolism in MASLD initiates hepatic fat accumulation, creating a critical foundation vulnerable to further liver damage and disease progression (Figure 5).

### 3.2 Insulin Resistance and Metabolic Syndrome

**FIGURE 6:**
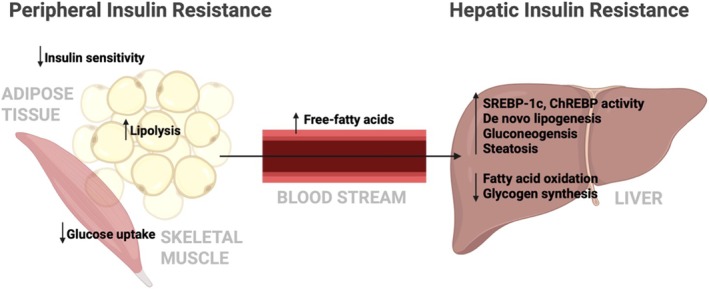
Interplay between peripheral and hepatic insulin resistance in MASLD pathogenesis. Peripheral insulin resistance increases circulation free fatty acids and hyperinsulinemia, which in turn drive hepatic insulin resistance, gluconeogenesis and lipogenesis. These processes promote steatosis, mitochondrial dysfunction and progression from MASLD to MASH.

Insulin resistance plays a central role in MASLD pathogenesis, disrupting hepatic glucose and lipid metabolism (Figure 5) [12]. In the liver, insulin resistance leads to increased gluconeogenesis and glucose production and reduced glycogen storage, exacerbating hyperglycemia and metabolic dysfunction [68, 69]. Peripheral insulin resistance, particularly in adipose and muscle tissues, results in compensatory hyperinsulinemia, which promotes hepatic lipid accumulation and impairs lipid oxidation [70]. This hormonal imbalance stimulates the overproduction of hepatic transcription factors: SREBP‐1c and ChREBP, which can promote the development of fatty liver through de novo lipogenesis [71, 72, 73, 74]. When coupled with impaired lipid oxidation and increased influx of free fatty acids, this metabolic environment favors steatosis. Over time, lipid overload and increased liver injury can result in oxidative stress and inflammatory signalling cascades, promoting the progression to steatohepatitis. Coexisting features of metabolic syndrome including obesity, dyslipidemia and hypertension intensify systemic and hepatic metabolic dysfunction. Thus, insulin resistance initiates and perpetuates hepatic fat accumulation and inflammation, linking systemic metabolic imbalances with the liver‐specific pathology that defines MASLD and MASH (Figure 6). Overall, insulin resistance acts as a driving force that connects systemic metabolic disturbances with hepatic lipid accumulation and inflammation, making it a key contributor to MASLD progression.

### 3.3 Mitochondrial Dysfunction and Oxidative Stress

Mitochondria maintain their functionality through dynamic processes of fission and fusion. In MASLD, this balance is disrupted, leading to excessive mitochondrial fragmentation or elongation, impacting their function and contributing to disease progression [75] (Figure 5). Studies indicate that disrupted mitochondrial dynamics impair cellular energy metabolism and exacerbate stress responses [76, 77, 78]. Furthermore, impaired mitophagy, the selective removal of damaged mitochondria, results in the accumulation of dysfunctional organelles, aggravating liver injury through triggering inflammation and fibrosis [79].

Mitochondria play a role in hepatic energy metabolism, and their dysfunction is a hallmark of MASLD progression, contributing to lipid accumulation, inflammation and fibrosis [1]. In MASLD, increased lipid oxidation and oxidative phosphorylation generate excessive reactive oxygen species (ROS), leading to mitochondrial damage and hepatocyte injury [80]. Additionally, mitochondrial dysfunction plays a direct role in liver fibrosis. ROS generated by dysfunctional mitochondria act as fibrogenic signals, activating hepatic stellate cells [81, 82]. These cells transition into a myofibroblast‐like state, driving excessive collagen deposition and extracellular matrix remodelling [83]. This dysfunction also impairs β‐oxidation, causing an accumulation of toxic lipid intermediates that drive lipotoxicity and cellular stress [80]. Additionally, mitochondrial abnormalities disrupt ATP production, impair autophagy and enhance apoptosis, further promoting hepatocyte injury and fibrosis [80].

Mitochondria are essential for cellular energy production through oxidative phosphorylation (OXPHOS), where ATP is generated. In MASH, OXPHOS efficiency is impaired, leading to reduced ATP production and increased ROS generation [77]. This imbalance exacerbates oxidative stress, creating a vicious cycle of mitochondrial damage. As ROS levels rise, they initiate lipid peroxidation and inflammatory signalling pathways, further contributing to hepatocyte injury and liver dysfunction [84] (Figure 5). Calcium homeostasis is critical for mitochondrial function and hepatocyte viability [85]. In MASLD, mitochondrial calcium overload disrupts metabolic processes, leading to ROS production and mitochondrial permeability transition pore (mPTP) opening [86, 87]. Calcium dysregulation represents a key pathological mechanism linking mitochondrial dysfunction to liver damage. The mPTP is a non‐selective channel that regulates mitochondrial membrane potential, and in pathological states like MASLD, its opening is triggered by calcium overload, oxidative stress and ATP depletion, which can lead to cell death [88]. This leads to loss of mitochondrial membrane potential, cytochrome c release and eventual hepatocyte death [89] (Figure 5). Intrinsic apoptosis is a key mechanism of hepatocyte death in MASH. Mitochondrial dysfunction facilitates cytochrome c release into the cytosol, activating caspases, proteases that dismantle the cell and drive apoptosis [82]. This process is closely linked to fibrogenesis, as the death of hepatocytes activates hepatic stellate cells, promoting collagen deposition and fibrosis [90]. The dual role of mitochondria in energy metabolism and apoptosis highlights their pivotal contribution to MASH pathogenesis.

Thus, mitochondrial dysfunction in MASLD and MASH contributes to a cascade of pathological processes, including impaired energy metabolism, oxidative stress, calcium imbalance and hepatocyte apoptosis, that collectively drive inflammation, fibrosis and disease progression.

### 3.4 Gut‐Liver Axis and Microbiome Alterations

Mitochondrial dysfunction in MASLD extends its impact beyond the liver; emerging evidence suggests that gut dysbiosis, alterations in intestinal barrier function and dysregulation in the gut‐liver axis contribute to MASLD pathogenesis [91, 92, 93]. An imbalance in gut microbiota composition can increase intestinal permeability, allowing bacterial endotoxins, such as lipopolysaccharides, to enter the portal circulation and trigger hepatic inflammation [94, 95]. Changes in bile acid metabolism and short‐chain fatty acid production further influence hepatic lipid metabolism and inflammatory pathways [96].

These microbiota‐driven changes amplify hepatic inflammation, influencing overall health and further driving MASLD progression. Compounding this, damaged mitochondria in hepatocytes release damage‐associated molecular patterns (DAMPs), which enter systemic circulation and trigger widespread inflammatory and immune responses [97]. Notably, disruption of the intestinal barrier acts synergistically with dysbiosis to facilitate entry of microbial products and metabolites into the liver, triggering hepatic immune activation and accelerating disease progression [98].

Current literature points to a bidirectional relationship between gut microbes and mitochondrial function in MASLD [99, 100, 101]. The gut microbiota influences mitochondrial pathways through the production of key metabolites that impact energy homeostasis, oxidative stress and immune signalling [98]. This microbiota‐mitochondrial crosstalk forms a potentially pathological loop in which microbial dysfunction impairs mitochondrial function, and mitochondrial injury in turn worsens gut barrier dysfunction and dysbiosis [98]. As such, mitochondria are now recognised as central targets through which the microbiota exerts effects on host physiology. Given this interconnected pathophysiology, therapeutic strategies targeting the gut mycobiome, including probiotics, prebiotics, dietary interventions, metabolic pathway targeting and faecal microbiota transplantation are gaining traction; these approaches aim to restore microbial balance, enhance barrier integrity and support mitochondrial function [99]. Therefore, this research is an area of growing interest for MASLD treatment.

Gut dysbiosis and impaired barrier function establish a self‐reinforcing cycle of mitochondrial injury and inflammation that drives MASLD progression. Targeting the gut microbiota with probiotics, prebiotics and specific dietary fibres has been shown to boost short‐chain fatty acid production, which in turn activates mitochondrial bioenergetics via PCG‐1α signalling, lowers hepatocellular ROS and suppresses NLRP3 inflammasome‐mediated cytokine release [102, 103]. Additionally, faecal microbiota transplantation can restore microbial diversity, enhance tight‐junction integrity and improve mitochondrial respiratory capacity in hepatocytes, collectively attenuating both local and systemic inflammatory responses. However, the durability of faecal microbiota transplantation's benefits remains unclear, as without sustained dietary or microbial support, microbial communities may revert to a dysbiotic state over time [104]. These interventions underscore the therapeutic promise, and current limitations, of modulating the gut–mitochondria–immune axis to ameliorate mitochondrial dysfunction and chronic inflammation in MASLD.

### 3.5 Inflammatory Pathways and Immune Response

Chronic low‐grade inflammation is a key factor in MASLD progression, mediated by innate immune activation and cytokine signalling. Kupffer cells, hepatic stellate cells and infiltrating macrophages contribute to a pro‐inflammatory environment through the release of cytokines such as tumour necrosis factor‐ α and interleukin‐1β, which are key players in liver inflammation and damage [105, 106]. Persistent inflammation leads to hepatocyte injury, activation of fibrogenic pathways and extracellular matrix deposition, ultimately resulting in fibrosis and cirrhosis [107].

### 3.6 MASH Pathology

**FIGURE 7:**
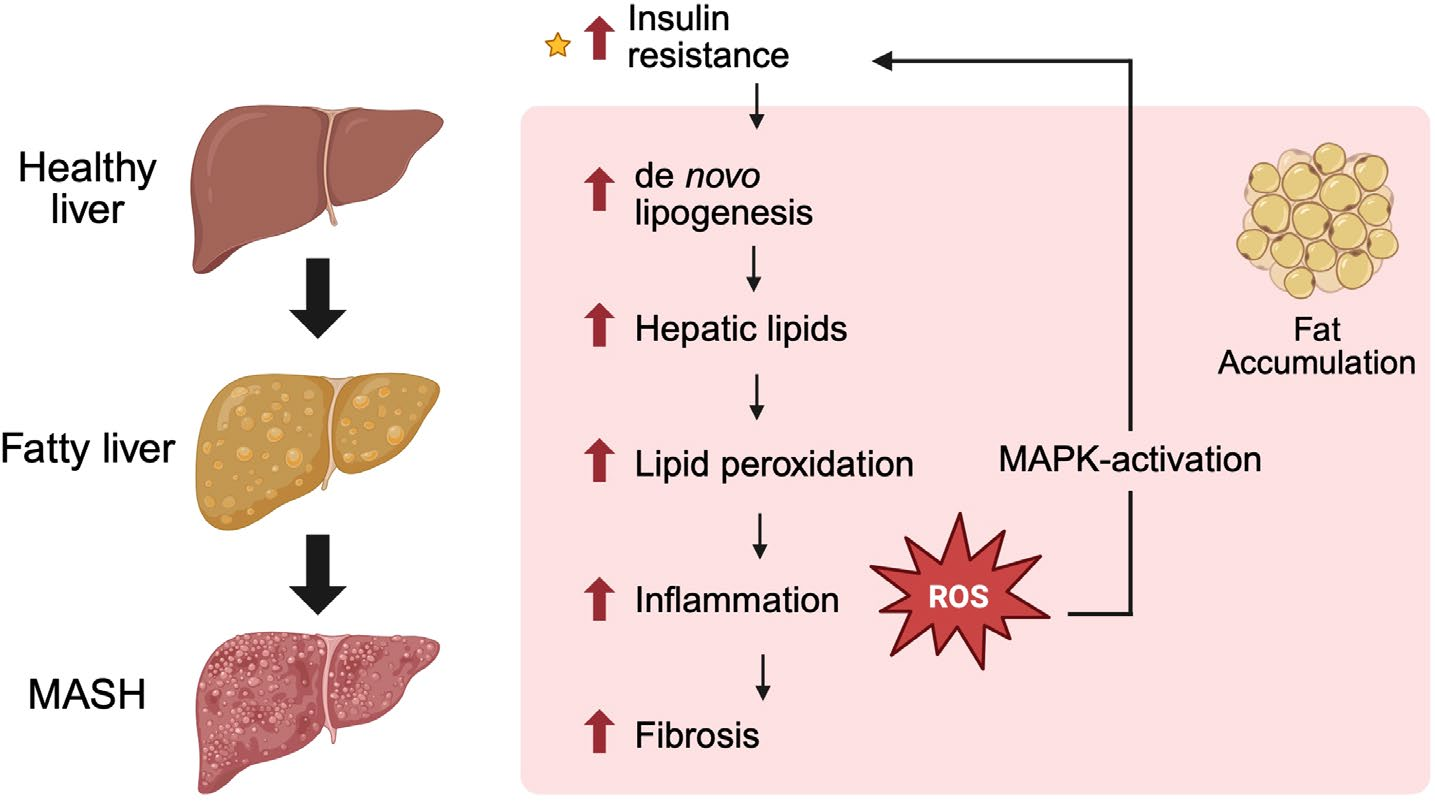
The multiple parallel‐hit hypothesis explains the progression of metabolic dysfunction‐ associated steatohepatitis (MASH). MASH progression is driven by insulin resistance, de novo lipogenesis and hepatic lipid accumulation, which promote lipid peroxidation and reactive oxygen species (ROS) generation. These insults contribute to mitochondrial injury and dysfunction, impairing oxidative phosphorylation (OXPHOS) and amplifying oxidative stress. Damaged mitochondria act as both sources and targets of cellular injury, further activating inflammatory pathways, including mitogen‐activated protein kinase (MAPK), and accelerating hepatocellular damage and fibrosis (adapted from Ota [109]).

MASH is a necroinflammatory liver disease governed by multiple pathways that are not entirely elucidated [108]. The pathology of MASH follows a complex progression, best explained by the ‘multiple parallel‐hit’ hypothesis [109, 110]. Factors like a high‐fat diet or insulin resistance initially lead to hepatic steatosis, where excess triglycerides, cholesterol and free fatty acids accumulate in the liver [109, 111]. This lipid overload promotes de novo lipogenesis and increases lipid peroxidation, which generates ROS and activates inflammatory pathways, specifically the mitogen‐activated protein kinase (MAPK) pathway. ROS and MAPK activation contribute to a cycle of oxidative stress and inflammation that damages liver cells [51, 109]. Insulin resistance and hyperinsulinemia‐driven de novo lipogenesis further exacerbate the process by continuously driving lipid accumulation in the liver, reinforcing the inflammatory response [109]. This inflammation recruits immune cells, such as Kupffer cells and M1 macrophages, which in turn activate hepatic stellate cells, ultimately leading to steatohepatitis [112, 113]. Over time, the liver transitions from a healthy state to fatty liver and eventually to MASH, marked by increased fibrosis and tissue damage [114] (Figure 7). This model highlights that MASH results from the combined effects of insulin resistance, lipid accumulation, oxidative stress and inflammatory pathways rather than a single triggering factor, explaining its varied risk profiles [109, 115].

### 3.7 Mitochondrial DNA and MASH

Variations in mtDNA and mitochondrial metabolism play a role in the progression of MASH, contributing to key pathological features such as lipid accumulation, insulin resistance and fibrosis [116, 117, 118, 119]. High rates of mtDNA mutations, particularly in OXPHOS genes, disrupt energy homeostasis, increase oxidative stress and amplify liver damage [119, 120]. Emerging evidence suggests that mtDNA epigenetic modifications, such as hypermethylation, also impair mitochondrial gene expression and metabolic activity, driving metabolic dysfunction in MASH [117]. For instance, in MASH patients, hypermethylation of the mitochondrial‐encoded *ND6* gene reduces its transcription and protein levels, impairing Complex I function and mitochondrial energy production [117, 121]. ROS‐driven oxidative damage to mtDNA and structural abnormalities in mitochondria create a vicious cycle of mitochondrial dysfunction and hepatocellular injury [19, 118]. mtDNA copy number variations may reflect a compensatory response to mitochondrial damage in MASLD/MASH, but this response eventually fails, leading to disease progression [122]. Furthermore, mtDNA deletions and mutations, such as those affecting cytochrome B, a critical component of the OXPHOS system, have been associated with advanced disease stages and greater inflammation [123].

Sookoian et al. [120] demonstrated that MASH patients exhibit a 1.28‐fold higher mtDNA mutation rate and increased heteroplasmy compared to controls, with disease severity correlating to a 1.4‐fold increase in mutations, particularly in genes involved in OXPHOS [120]. These mutations disrupt gene and protein expression, with OXPHOS polymorphic sites linked to more severe MASLD phenotypes. A notable missense mutation in the POLG gene (p.Gln1236His) has been associated with changes in liver mtDNA copy numbers, further emphasising mtDNA's role in MASH [120]. Additionally, OXPHOS genes exhibit mutation hotspots tied to severe phenotypes, potentially originating from a shared germline source [120]. Structural and functional mitochondrial defects, including reduced respiratory chain activity, impaired β‐oxidation and ultrastructural lesions, further drive the transition from steatosis to severe liver damage [119]. This evidence highlights abnormalities of mtDNA as a central factor in MASH progression and a promising target for therapeutic interventions aimed at mitigating its systemic and hepatic impacts [19].

In addition, Betancourt et al. provide insights into the interaction between mitochondrial DNA (mtDNA) and diet in the context of MASLD/MASH. Using Mitochondrial‐Nuclear eXchange (MNX) mice to isolate mtDNA effects from nuclear DNA (nDNA) effects, they demonstrated that mitochondrial and nuclear genomic interactions significantly influence diet‐induced MASLD progression [19]. When fed an atherogenic diet (TD.02028, 21% fat, 1.25% cholesterol, 0.5% cholic acid), mice with the cardiometabolic disease‐prone (C57BL/6J) nuclear genome developed more severe macrosteatosis, inflammation and fibrosis compared to those with the cardiometabolic disease‐resistant (C3H/HeN) nuclear genome. These effects were associated with distinct changes in mitochondrial function, including increased State 4 respiration and altered expression of mitochondrial biogenesis genes [19]. Additionally, the study revealed that the cardiometabolic disease‐prone nuclear genome was associated with increased Complex IV activity and elevated expression of genes involved in inflammation and fibrosis. Betancourt et al. demonstrated that the C57BL/6J nuclear genome was a major driver of hepatic inflammation and fibrosis, while the mitochondrial genome contributed to lipid metabolism and mitochondrial respiratory function. Mice with C57^nDNA^ exhibited more severe macrosteatosis, inflammatory infiltration and collagen deposition, and MNX mice developed intermediate phenotypes, suggesting that mito‐nuclear interactions are key in determining liver injury [19].

### 3.8 Understanding Disease Heterogeneity

One of the major challenges in MASLD research and treatment is understanding disease heterogeneity. MASLD is a multifaceted condition influenced by genetic predisposition, environmental factors and metabolic variations [124]. Genetic susceptibility differs among populations, with certain ethnic groups, such as individuals of Hispanic descent, displaying a higher risk due to variations in genes such as *PNPLA3* [125]. Additionally, the presence of distinct metabolic phenotypes within MASLD, including those with insulin resistance‐driven disease [56] and those with lean MASLD [126], highlights the need for tailored diagnostic and therapeutic approaches. Identifying subtypes of MASLD based on these factors is crucial for improving patient stratification and treatment outcomes.

## 4 Genetics

### 4.1 Genetic Aetiology

MASLD is a multifactorial disorder with a complex genetic foundation that influences susceptibility, disease progression and the risk of advanced liver pathology. Although lifestyle, diet and metabolic comorbidities are primary drivers of disease onset, genetic variation critically modulates an individual's risk of developing steatosis, progression to inflammation and fibrosis or maintaining liver health despite similar metabolic stressors. Landmark genome‐wide association studies have identified several commonly occurring variants with significant effects on hepatic lipid metabolism and injury response.

**FIGURE 8:**
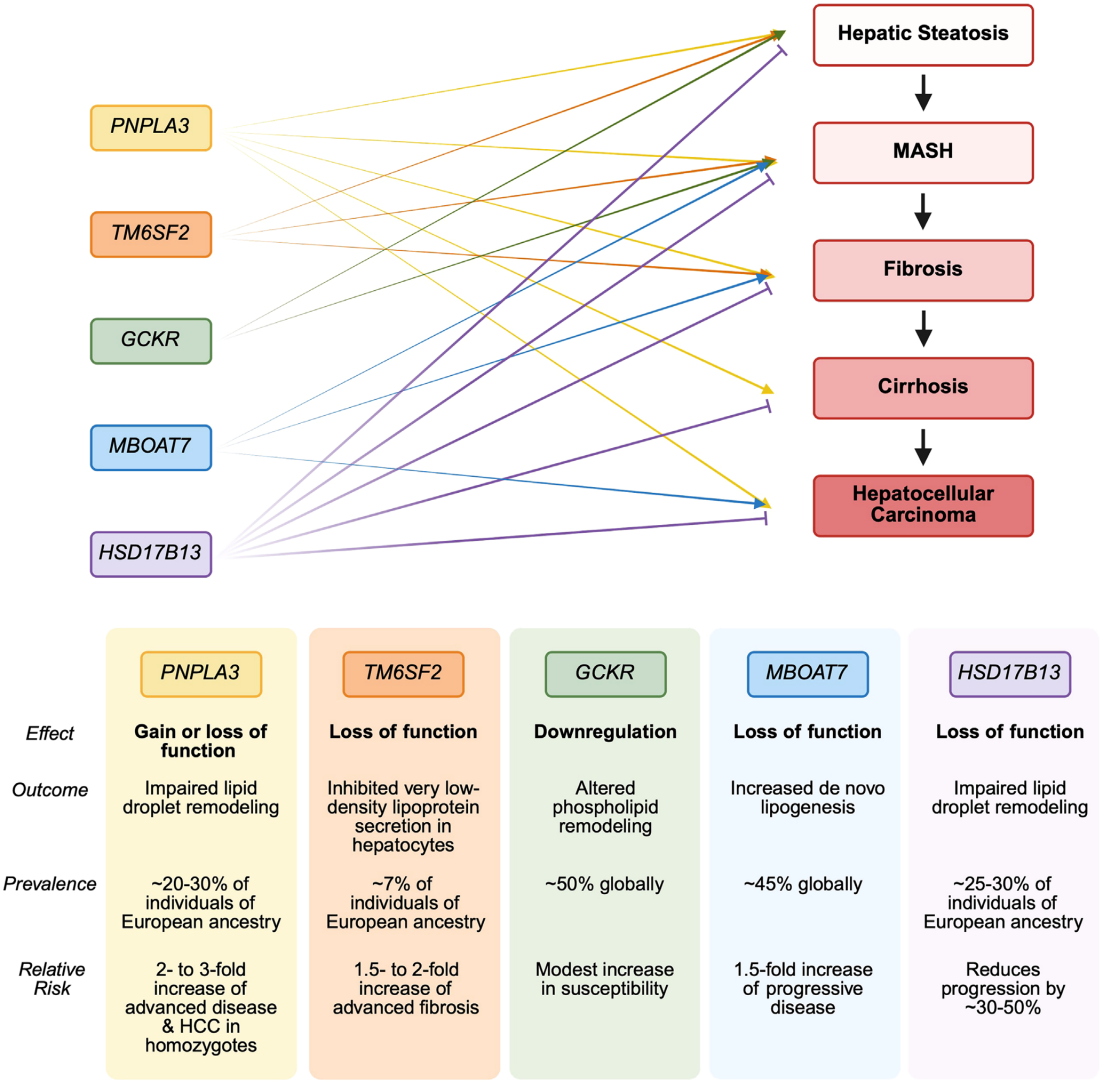
Frequently studied genetic variants implicated in the pathogenesis of metabolic dysfunction‐associated steatotic liver disease (MASLD). Several gene variants have been associated with MASLD susceptibility, but key contributors with varying prevalence such as PNPLA3, TM6SF2, GCKR, MBOAT7 and HSD17B13, are among the most well‐characterised and strongly linked to disease progression. These variants impair hepatic lipid metabolism and have differing impacts on hepatic steatosis, metabolic dysfunction‐associated steatohepatitis (MASH), fibrosis, cirrhosis and hepatocellular carcinoma (HCC) conferring varying relative risk [128].

The strongest known genetic determinant of MASLD is the rs738409 C>G polymorphism in the patatin‐like phospholipase domain‐containing 3 (*PNPLA3*) gene on chromosome 22 [127]. This missense variant (I148M) impairs triglyceride hydrolysis in hepatocytes, promoting intracellular lipid retention; it also correlates with a more severe manifestation of all stages in MASLD progression (Figure 8). The I148M allele is most prevalent in Hispanic populations, intermediate in Europeans and least common in individuals of African descent, mirroring global MASLD epidemiology [129]. Several other genes exert important influences. The transmembrane 6 superfamily member 2 (*TM6SF2*) rs58542926 C>T (E167K) variant reduces very low‐density lipoprotein secretion, exacerbating hepatic fat storage while paradoxically lowering circulating lipids [130] (Figure 8). The glucokinase regulatory protein (*GCKR*) rs1260326 C>T (P446L) variant augments glucose‐driven de novo lipogenesis, and the membrane bound O‐acyltransferase domain‐containing 7 (*MBOAT7*) rs641738 C>T allele upsets phospholipid remodelling, both contributing to steatosis and inflammation [131, 132]. In contrast, an insertion in 17‐β hydroxysteroid dehydrogenase 13 (*HSD17B13*) rs72613567 T>A appears protective, mitigating progression from steatohepatitis to HCC even among high‐risk *PNPLA3* carriers [133]. Collectively, these and other loci form the basis of emerging polygenic risk scores, which better stratify individuals, and contribute to explaining why ‘lean MASLD’ can occur in genetically susceptible individuals with normal BMI. These variants are not exhaustive but represent the most frequently studied and clinically relevant genetic contributors to MASLD risk.

### 4.2 Molecular Genetic Sequencing in Diagnostics

While liver biopsy and imaging remain diagnostic gold standards, genotyping high‐impact variants (*PNPLA3* I148M, *TM6SF2* E167K) via targeted PCR or inclusion in next‐generation sequencing panels holds promise for early risk assessment. Genetic risk information may guide surveillance intensity in atypical patients (e.g., normal‐weight steatosis), augment non‐invasive fibrosis algorithms (FIB‐4, NAFLD fibrosis score), and ultimately inform personalised therapies now under development for specific genetic subtypes [128, 134]. However, despite this potential, routine genetic testing in MASLD is not yet established. Interpretation of risk alleles requires integration with clinical, metabolic and environmental data, and the cost–benefit balance awaits validation in the prospective cohorts [135].

### 4.3 Genetic Counselling

Given MASLD's polygenic and modifiable nature, genetic counselling is not universally indicated. While heritability estimates for MASLD are high, with studies showing unadjusted heritability up to 0.85 and adjusted estimates for liver fat fraction ~0.39, indicating a substantial genetic contribution, this reflects the combined effects of many variants rather than rare monogenic causes [136]. Thus, strongly penetrant genetic forms of MASLD remain relatively uncommon, comprising a small minority of cases. However, in select scenarios (early‐onset disease, unexplained progressive fibrosis, cryptogenic cirrhosis or strong family histories of cirrhosis/HCC), knowledge of high‐risk alleles may prompt earlier lifestyle interventions and tailored monitoring [137, 138, 139]. First‐degree relatives of affected individuals, particularly in high‐prevalence ancestries (e.g., Hispanic populations with high *PNPLA3* I148M frequency), may also benefit from risk stratification [140]. In paediatric populations, understanding a child's genetic risk may justify earlier lifestyle counselling and incorporation of liver‐protective nutrients into the diet [141]. Ultimately, MASLD counselling bridges genetic insight with actionable prevention strategies, emphasising that genotype informs risk but does not determine outcome.

## 5 Diagnosis

### 5.1 Diagnostic Criteria and Non‐Invasive Biomarkers

Simple steatosis is largely asymptomatic and is characterised by macrovesicular steatosis with minimal or no inflammation. However, it has the potential to progress if metabolic dysfunction is not managed [142]. In contrast, MASH presents with more severe histological evidence, including hepatocyte ballooning, lobular inflammation, apoptotic bodies and the presence of Mallory‐Denk bodies (MDB) [42]. Fibrosis is often observed but is not required for diagnosis; however, the presence of fibrosis is associated with worse prognosis [42]. Notably, the histological changes associated with MASH are most pronounced in acinar zone 3, and their distribution varies between biopsies, making diagnosis complex [42]. Although non‐invasive methods are improving, liver biopsy continues to be the definitive approach for differentiating MASH from simple steatosis, especially when imaging or biomarker findings are unclear [51]. Histological confirmation of MASH typically includes features such as hepatocellular ballooning, lobular inflammation, MDB and various stages of fibrosis [51].

While liver biopsy and histopathological examination of MASLD are essential for distinguishing between simple steatosis and MASH, they are increasingly being complemented or replaced by non‐invasive biomarkers and imaging modalities. For example, MASLD diagnosis often begins with abnormal liver function tests or imaging techniques that identify hepatic steatosis. Ultrasound remains the primary screening tool due to its accessibility; however, it lacks sensitivity in mild cases. More specific imaging techniques being used include magnetic resonance imaging proton density fat fraction (MRI‐PDFF) which is a highly accurate modality for quantifying liver fat, commonly used in clinical trials and transient elastography (FibroScan) which measures liver stiffness and provides an indirect assessment of fibrosis severity, aiding in risk stratification [45].

Serum biomarkers offer additional diagnostic utility. Commonly used indicators include alanine aminotransferase (ALT) and aspartate aminotransferase (AST), as they are both commonly elevated in MASLD, though their sensitivity is limited [143, 144]. More specialised biomarkers, such as cytokeratin‐18 (CK‐18: a marker of hepatocyte apoptosis and necrosis), reflect hepatocellular injury and are useful in distinguishing MASH from simple steatosis, while γ‐glutamyl transferase (GGT) has been associated with advanced fibrosis and mortality risk [145].

Despite these advancements, challenges persist. Many non‐invasive diagnostics and tools struggle to differentiate between MASLD and other liver diseases, as well as their underlying etiologies [146]. Additionally, non‐invasive tests often cannot distinguish between intermediate disease stages or detect early hepatocyte injury and fibrosis [146]. There is an increasing need for research that defines serum scores with MASLD characteristics and the temporal kinetics of the disease. Variability in results across patient populations and comorbid conditions also complicates the interpretation of non‐invasive diagnostics [146]. Emerging diagnostic approaches including lipidomic and metabolomic profiling [147], microRNA‐based signatures [148], imaging tools like magnetic resonance elastography (MRE) [149] and combinations of various scores and tools may improve the specificity, sensitivity and accuracy of disease staging while minimising patient burden.

### 5.2 Risk Stratification and Clinical Scoring Systems

Given the progressive and complex nature of MASLD, clinical scoring systems play a pivotal role in patient management, helping to standardise the assessment of disease severity. These scoring methods measure the extent of steatosis, inflammation and fibrosis to aid diagnosis and disease monitoring. Area Under the Receiver Operating Characteristic Curve (AUROC) is used to evaluate the accuracy of non‐invasive tests and scores in diagnosing and staging liver conditions [150].

#### 5.2.1 Fibrosis‐Focused Tools

Among the most validated and widely used tools are: (i) FIB‐4 Index and (ii) NAFLD Fibrosis Score (NFS)—widely used for non‐invasive fibrosis assessment and prioritisation of high‐risk patients for specialist referral (Table 2).

**TABLE 2:** Summary of MASLD clinical scoring systems.

Category	Score name	AUROC	Key components	MASLD validation	Source(s)
Steatohepatitis‐focused tools	oxNASH	0.72–0.74	13‐hydroxyl‐octadecadienoic acid/linoleic acid (oxFA) ratio, AST, age, BMI	No	Feldstein, Lopez et al. [151], Alkhouri, Berk et al. [152], Abdelhameed, Kite et al. [153]
	Palekar score	0.76	AST, AST/ALT ratio, hyaluronic acid, BMI, age	No	Palekar, Naus et al. [154], Abdelhameed, Kite et al. [153]
	NAFIC score	0.78–0.85	Insulin, ferritin, type IV collagen 7 s	No	Sumida, Yoneda et al. [155], Abdelhameed, Kite et al. [153]
	NashTest	0.79	Age, sex, weight, height, GGT, ALT, AST, serum triglycerides, cholesterol, apolipoprotein A, α2‐macroglobulin, haptoglobin, total bilirubin	No	Dixon, Bhathal et al. [156], Sumida, Yoneda et al. [155], Abdelhameed, Kite et al. [153]
	HAIR model	0.90	Hypertension, increased ALT, insulin resistance	No	Dixon, Bhathal et al. [156], Abdelhameed, Kite et al. [153]
Steatosis‐focused tools	SteatoTest	0.80–0.82	ALT, GGT, glucose, age, gender, BMI, cholesterol, α2‐macroglobulin, apolipoprotein A‐I, haptoglobin, total bilirubin, triglycerides	No	Poynard, Ratziu et al. [157], Abdelhameed, Kite et al. [153]
	NAFLD liver fat score	0.80–0.87	FSI, AST and AST/ALT Ratio, Metabolic syndrome, type 2 diabetes	Yes	Kotronen, Peltonen et al. [158], Abdelhameed, Kite et al. [153]
	Hepatic steatosis index (WHICH)	0.68–0.81	BMI, AST/ALT ratio, diabetes	Yes	Lee, Kim et al. [159], Abdelhameed, Kite et al. [153]
	Fatty Liver Index (FLI)	0.70–0.84	BMI, GGT, waist circumference, triglycerides	Yes	Bedogni, Bellentani et al. [160], Abdelhameed, Kite et al. [153]
Fibrosis‐focused tools	FIB‐4 index	0.80–0.82	AST, ALT, Age, Platelets	Yes	Mcpherson, Hardy et al. [161], Abdelhameed, Kite et al. [153]
	BARD score	0.59–0.81	BMI > 28, AST/ALT > 0.8, diabetes	No	Harrison, Oliver et al. [162], Abdelhameed, Kite et al. [153]
	APRI	0.75–0.85	AST, platelets	Yes	Xiao, Zhu et al. [163], Abdelhameed, Kite et al. [153]
	NFS	0.68–0.92	Age, BMI, glucose, AST/ALT, albumin, platelets	Yes	Angulo, Hui et al. [164], Abdelhameed, Kite et al. [153]
Emerging models	MLA score	0.89	PRO‐C3, collagen, AST/GGT, BMI	No	Feng, Zheng et al. [165], Abdelhameed, Kite et al. [153], Dabbah, Mishani et al. [166]
	Enhanced liver fibrosis (ELF)	0.80–0.83	Age, hyaluronic acid, PIIINP, TMP1	No	Lichtinghagen, Pietsch et al. [167], Abdelhameed, Kite et al. [153]
	MASLD liver fat score (LFS)	N/A	Machine learning, metabolic syndrome, type 2 diabetes, FSI, AST and AST/ALT ratio	Yes	Kotronen, Peltonen et al. [158], Abdelhameed, Kite et al. [153]
	MASLD ridge score	N/A	Machine learning, Type 2 Diabetes, hepatitis risk score, ALT, triglycerides, HDL‐C	Yes	Abdelhameed, Kite et al. [153], Diaz, Arab et al. [168]
	ADAPT score	0.88	PRO‐C3, AST, platelets	No	Eslam, Wong et al. [169], Abdelhameed, Kite et al. [153]

*Note:* A categorization of established and emerging clinical scoring systems used to assess steatosis, steatohepatitis and fibrosis in MASLD. For each tool, the diagnostic performance (AUROC range), core components and validation status in MASLD‐specific populations are presented.

Abbreviations: ALT, Alanine Aminotransferase; AST, Aspartate Aminotransferase; AUROC, Area Under the Receiver Operating Characteristic Curve; BMI, Body Mass Index; FSI, Fasting Serum Insulin; PIIINP, Procollagen III N‐terminal peptide; PRO‐C3, N‐terminal propeptide of type III collagen; TMP1, Tissue Inhibitor of Metalloproteinase 1.

A crucial step in managing MASLD is identifying patients with significant fibrosis, as this subgroup faces the highest risk of liver‐related complications. The FIB‐4 index is a commonly used non‐invasive tool that estimates liver fibrosis severity based on age, liver enzyme levels (AST and ALT) and platelet count [170]. It has shown consistent diagnostic performance in MASLD patient populations with an AUROC ranging from 0.80 to 0.82 [153]. The index is divided as follows: F0–F1—minimal fibrosis; low risk of progression, F2–F3—intermediate fibrosis; requires close monitoring, F4 (cirrhosis)—high risk of liver decompensation and HCC, considered for screening programs and evaluation for clinically significant portal hypertension. Similarly, the NFS incorporates clinical parameters such as age, BMI, glucose levels, AST/ALT, albumin and platelets to predict fibrosis risk [164]. In comparison, transient elastography and MR elastography provide accurate fibrosis staging, and scoring systems (like FIB‐4 and NFS) serve as practical, non‐invasive alternatives in resource‐limited settings [171]. Composite scoring systems, such as the FIB‐4 index and NFS, aid in risk stratification by integrating clinical parameters like age, liver enzymes and metabolic factors [170, 172].

#### 5.2.2 Steatosis‐Focused Tools

Scoring systems used to detect steatosis in MASLD patient populations include the Hepatic Steatosis Index (WHICH), Fatty Liver Index (FLI), SteatoTest and the NAFLD Liver Fat Score (Table 2). Steatosis‐focused tools usually encompass anthropometric measurements, lipid profiles and liver enzymes.

The WHICH utilises liver enzyme ratios and BMI to assess the likelihood of hepatic steatosis and has moderate diagnostic accuracy in MASLD with an AUROC ranging from 0.68 to 0.81 [153, 172]. However, Abdelhameed et al. highlight limitations related to diagnostic accuracy in children with obesity, as well as a reduced ability to distinguish stages of steatosis [153]. The FLI has been validated in MASLD, with an AUROC ranging from 0.70 to 0.84, but concerns regarding its ability to distinguish the severity of steatosis remain [153]. The SteatoTest combines metabolic and biochemical markers for steatosis evaluation, and although validated in steatosis and NAFLD patients, it has yet to be validated in MASLD populations [153]. The NAFLD Liver Fat Score utilises diagnostic markers such as FSI, AST and AST/ALT ratio, Metabolic Syndrome characteristics, Type 2 diabetes mellitus, and has been validated in MASLD populations with an AUROC ranging from 0.80 to 0.87 [153, 158]. While this tool has the benefit of being reliable and easy to use, it is costly and requires measuring serum fasting insulin levels [153].

#### 5.2.3 Steatohepatitis‐Focused Tools

Identifying and grading patients with steatohepatitis presents challenges; however, several specialised scores have been proposed (Table 2). The Hypertension, ALT, Insulin Resistance (HAIR) Model demonstrates excellent diagnostic performance in obesity cohorts with an AUROC of 0.90, although validity in MASLD populations is lacking and its high associative costs remain a challenge [153]. Other tools such as the oxNASH, Palekar Score, NAFIC Score and the NashTest use combinations of inflammatory markers, metabolic data, fibrosis indicators and classic liver markers like AST/ALT to differentiate between simple steatosis and steatohepatitis [156]. Many of these tools show moderate AUROC values, ranging from 0.72 to 0.85; their clinical utility is limited by their reduced sensitivity, small validation cohorts and lack of validation in MASLD‐specific populations [153].

#### 5.2.4 Emerging Tools

More advanced models exist, such as the MASLD Liver Fat Score and MASLD Ridge Score, that incorporate machine learning, high‐dimensional data and metabolic profiling to enhance diagnostic precision [173] (Table 2). Additionally, the Enhanced Liver Fibrosis (ELF) panel, which measures age, hyaluronic acid, PIIINP and TMP1, has shown good diagnostic accuracy in identifying advanced fibrosis [153, 167]. Additional models like the ADAPT Score and MLA Score integrate biomarkers like PRO‐C3 and collagen breakdown products to uncover fibrogenesis, a known pathological process in MASLD [174]. These biomarkers specifically reflect extracellular matrix remodelling, which occurs during the progression from simple steatosis to fibrosis [174]. The novelty of these scores lies inherently in their ability to provide a dynamic assessment of fibrotic activity rather than solely relying on indirect indicators of liver injury. A study by Daniels et al. concluded that the ADAPT Score was superior to previously established fibrosis‐focused tools in identifying hepatic fibrosis in MASLD patients [153, 175]. However, further validation studies of these emerging tools in MASLD‐specific populations are necessary to establish their widespread clinical applicability.

However, with these scoring systems come limitations. Some common challenges include limited accuracy in early disease stages, variable performance across diverse populations, high costs and the need for further validation in MASLD‐specific populations [153].

#### 5.2.5 Genetic and Multi‐Omic Markers

There are also emerging genetic and proteomic markers such as: (i) PNPLA3 and TM6SF2 polymorphisms: genetic variants associated with increased risk of fibrosis and HCC and (ii) proteomic and metabolomic approaches: novel biomarkers are being explored for early detection and personalised risk prediction.

### 5.3 Need for Better Non‐Invasive Diagnostic Tools

Another pressing challenge in managing chronic liver diseases is the need for better development of non‐invasive diagnostic tools. While imaging techniques such as transient elastography (FibroScan) and MRI‐based assessments have improved diagnostic accuracy, there remains a strong demand for more accessible and precise methods. Emerging liquid biopsy markers, including circulating cell‐free DNA and extracellular vesicles, hold promise for detecting disease progression and fibrosis staging [176]. Additionally, artificial intelligence (AI)‐based imaging techniques are being developed to enhance the accuracy of fibrosis assessment and steatosis quantification [177]. The integration of multi‐omics approaches, including proteomics, metabolomics and transcriptomics, may further improve patient classification and enable earlier detection of high‐risk individuals.

To address these diagnostic gaps, several targeted research strategies are emerging. Advanced liquid biopsy techniques, such as EPIC‐Seq (Epigenetic Expression inferred by Cell‐free DNA Sequencing), are being investigated for their ability to infer tissue‐specific gene expression from circulating DNA, offering a powerful, minimally invasive approach to disease monitoring and staging [178]. Meanwhile, AI‐enhanced imaging is showing significant promise. For example, the European Medicines Agency recently approved AIM‐NASH, an artificial intelligence‐based histological analysis tool, for use in clinical trials aimed at evaluating MASH, highlighting its clinical relevance in standardising fibrosis assessment [179]. Researchers are also developing hybrid imaging models that combine ultrasound data and routine blood tests using machine learning classifiers, achieving high accuracy in staging liver fibrosis and cirrhosis [180]. Non‐contrast MRI techniques, when integrated with fully automated deep learning models, have also demonstrated superior performance in fibrosis staging compared to traditional modalities [181]. Furthermore, multi‐omics strategies continue to evolve as a frontier in patient stratification and early disease detection. Combining proteomic profiling with transcriptomics has outperformed conventional clinical biomarkers in early‐stage liver fibrosis detection [182]. Studies examining hepatic stellate cells through integrated omics have uncovered molecular signatures of advanced fibrosis, aiding in both diagnostic and therapeutic innovation [183].

Importantly, many of these innovations are moving from bench to bedside. Ongoing clinical studies are validating AI‐driven diagnostic platforms and multi‐omics biomarkers in real‐world patient cohorts, aiming to refine non‐invasive workflows and therapeutic monitoring strategies [184, 185]. Together, these advancements represent a concerted shift towards precision diagnostics that are scalable, reliable and capable of transforming clinical management of MASLD and other chronic liver diseases.

## 6 Treatment

### 6.1 Lifestyle Modifications as the Cornerstone of Treatment

Currently, two FDA‐approved pharmacological therapies exist for MASLD or MASH, while none are approved by Health Canada (discussed in Section 2.2). However, those that are approved are restricted to certain populations; thus lifestyle modifications remain the cornerstone of treatment, with dietary changes, exercise and weight loss forming the foundation of management strategies [51] (Table 3).

**TABLE 3:** Effectiveness of lifestyle interventions in reducing hepatic steatosis in MASLD.

Intervention	Mechanism	Reduction in hepatosteatosis	Additional benefits	Level of evidence
Mediterranean Diet [186, 187]	Anti‐inflammation, high MUFAs, low refined sugar	↓ 25%–35%	Improved insulin sensitivity, better lipid profile	High
Low‐Carbohydrate/Ketogenic Diet [188, 189, 190]	↓ De novo lipogenesis, ↑ fat oxidation	↓ 20%–40% (short‐term)	Rapid weight loss, ↓ triglycerides	Moderate to high
Low‐Fat Diet [191, 192]	↓ Caloric intake, ↓ dietary fat	↓ 10%–25%	Modest weight loss, improved LDL cholesterol	Moderate
Intermittent Fasting [193, 194, 195]	Caloric restriction	↓ 20%–30%	May reduce ALT/AST, improve insulin sensitivity	Emerging
Aerobic Exercise [196, 197]	↑ Fat oxidation, ↓ visceral fat	↓ 20%–30%	↑ Cardiorespiratory fitness, ↓ liver enzymes	High
Resistance Training [198, 199]	↑ Muscle mass, ↑ insulin sensitivity	↓ 10%–20%	Preserves lean mass	Moderate
Combined Aerobic + Resistance [24, 200]	Synergistic metabolic effects	↓ 30%–40%	Superior metabolic and functional improvements	High

*Note:* Compare and contrast various lifestyle interventions, including dietary patterns, fasting regimens and exercise modalities, based on their proposed mechanisms of action, estimated reduction in hepatic fat, associated metabolic benefits and the strength of supporting evidence in managing metabolic dysfunction‐associated steatotic liver disease (MASLD).

Dietary interventions such as the Mediterranean diet, which emphasises polyunsaturated fats, polyphenols and omega‐3 fatty acids, have been shown to improve liver histology and metabolic parameters [201]. A two‐year Mediterranean diet intervention demonstrated significant improvements in liver health among patients with MASLD, including reductions in liver fat and improvements in liver enzyme levels [202]. Low‐carbohydrate, high‐protein diets have also demonstrated benefits in reducing hepatic fat accumulation and improving insulin sensitivity [203, 204]. A recent study found that both low‐carbohydrate and low‐calorie diets significantly reduced liver fat content and improved brain metabolites associated with MASLD, suggesting potential cognitive benefits alongside hepatic improvements [205]. Intermittent fasting has emerged as another promising dietary strategy. A 12‐week randomised controlled trial comparing intermittent calorie restriction with continuous calorie restriction found that both approaches effectively reduced liver fat content, suggesting that intermittent calorie restriction can be an effective alternative for treating MASLD [206]. Additionally, a study combining time‐restricted feeding with flaxseed supplementation observed significant improvements in BMI, waist circumference, lipid and glycemic profiles, liver enzymes and inflammation markers in patients with MASLD [207].

Exercise also plays a crucial role, with both aerobic and resistance training proving effective in decreasing hepatic steatosis. Aerobic exercises enhance cardiovascular fitness and insulin sensitivity [208], while resistance training contributes to reductions in inflammatory markers and improved muscle mass [209]. Importantly, the benefits of diet and exercise interventions are often linked to the degree of weight loss achieved. A weight loss of at least 7%–10% has been identified as a critical threshold for reversing fibrosis and achieving meaningful histological improvements in MASLD patients [210]. These findings underscore the importance of personalised dietary and exercise interventions in managing MASLD.

### 6.2 Pharmacological Therapies

Despite lifestyle modifications being the first‐line approach, pharmacological therapies are increasingly being explored as adjunctive or alternative treatments for MASLD (Table 4). Given the complexity of MASLD pathogenesis, future research is focused on targeted therapies addressing metabolic dysfunction, inflammation and fibrosis progression [217]. Insulin sensitizers, such as pioglitazone, a peroxisome proliferator‐activated receptor gamma (PPAR‐γ) agonist, have shown promise in improving liver histology and reducing fibrosis [211]. Glucagon‐like peptide‐1 (GLP‐1) receptor agonists, including semaglutide and liraglutide, have demonstrated significant reductions in hepatic fat content and favorable metabolic effects in clinical trials [213], and semaglutide gained FDA approval in August 2025 for the treatment of adults with MASH and moderate‐to‐advanced fibrosis [218]. While GLP‐1s are among the most promising emerging therapies, potential drawbacks include lean muscle mass loss and rebound weight gain following treatment discontinuation [219, 220]. Thus, their clinical utility reflects a balance between substantial histological and metabolic benefits and the need to mitigate risks associated with long‐term use and discontinuation.

**TABLE 4:** Overview of pharmacological agents in clinical development for MASLD.

Class	Agent(s)	Mechanism of action	Current trial/approval status	Primary endpoint
Insulin Sensitizers [211, 212]	Pioglitazone	PPAR‐γ agonist; enhances insulin sensitivity, reduces hepatic lipogenesis and inflammation	Phase III trials; shown histological improvements in NASH	Resolution of MASH without worsening fibrosis
GLP‐1 Receptor Agonist [213]	Semaglutide, Liraglutide	Enhances insulin secretion, reduces appetite and body weight, decreases hepatic fat	Phase II‐III trials; semaglutide is FDA approved as of August 2025	Resolution of NASH without worsening fibrosis
SGLT2 Inhibitors [214]	Empagliflozin, Dapagliflozin	Inhibits renal glucose reabsorption; improves insulin sensitivity and reduces fat content	Phase III trials; promising reductions in hepatic steatosis	Improvement in liver fat content or ALT
Thyroid Hormone Receptor‐β Agonists [215]	Resmetirom (MGL‐3196)	Selectively activates liver‐specific thyroid hormone receptors to increase fat oxidation	FDA approved as of March 14, 2024	Resolution of MASH or improvement in fibrosis; reduction in liver fat content
Pan‐PPAR Agonists [216]	Lanifibranor	Activates PPAR‐α, ‐δ and ‐γ; addresses lipid metabolism, inflammation and fibrosis	Phase III ongoing	Improvement in fibrosis by ≥ 1 stage without worsening of MASH *or* MASH without worsening of fibrosis

*Note:* Summary of key classes of pharmacological agents currently under investigation for the treatment of metabolic dysfunction‐associated steatotic liver disease (MASLD), detailing their mechanisms of action and status of ongoing clinical trials. Agents include insulin sensitizers, incretin‐based therapies and nuclear modulators, each targeting distinct pathways involved in hepatic steatosis, inflammation and fibrosis.

Additionally, sodium‐glucose cotransporter‐2 (SGLT2) inhibitors, such as empagliflozin and dapagliflozin, have shown potential in improving insulin sensitivity and reducing hepatic steatosis [221]. Farnesoid X receptor (FXR) agonists, target bile acid metabolism and have been investigated for their potential to reduce liver fibrosis and inflammation [222]. Most notably, Resmetirom, a thyroid receptor‐β agonist, became the first FDA‐approved therapy for non‐cirrhotic MASH with moderate to advanced fibrosis in March 2024, marking a major milestone in the pharmacological treatment of the disease [223].

### 6.3 Addressing Socioeconomic Disparities in MASLD Management

Addressing socioeconomic disparities in MASLD management is also imperative, as access to healthcare remains a significant barrier for many affected individuals. Low‐income populations and marginalised communities often face difficulties in obtaining early diagnoses and receiving appropriate medical care [224]. Limited access to specialised diagnostic tools, expensive pharmacotherapies and nutritional counselling, coupled with food insecurity exacerbates health inequities [225]. Public health policies and community‐based interventions play a vital role in bridging these gaps by promoting awareness, subsidising healthcare costs and implementing screening programs in high‐risk populations [226]. Ensuring equitable access to MASLD treatment and prevention strategies is essential for reducing the overall disease burden and improving patient outcomes.

A cohort study conducted in southern Italy examined the relationship between educational attainment and the prevalence of MASLD. The study found a strong inverse association between education level and MASLD risk. Specifically, individuals with middle school education have an odds ratio of 0.50, high school graduates have an odds ratio of 0.29, and those with graduate education have an odds ratio of 0.24, all indicating a significantly lower risk compared to those with lower educational levels [227]. These findings suggest that lower education attainment, which often correlates with reduced health literacy and limited access to healthcare resources, may contribute to higher MASLD prevalence in certain populations [227]. This underscores the importance of addressing education and socioeconomic factors in MASLD prevention and management strategies.

Looking ahead, future research should prioritise understanding MASLD subtypes, refining diagnostic methodologies, advancing precision medicine and addressing healthcare disparities. With continued advancements in genetics, artificial intelligence and therapeutic development, the landscape of MASLD management is set up for significant transformation. Implementing community‐based education programs and ensuring equitable access to healthcare services are vital steps towards mitigating the impact of socioeconomic disparities on MASLD outcomes. A multidisciplinary approach that integrates clinical, molecular and socioeconomic perspectives will be essential for effectively tackling the complexities of this disease and improving long‐term patient care.

## 7 Treatments in Development

### 7.1 Advancing Personalised Medicine

The advancement of personalised medicine is another critical area of focus in MASLD management. Given the variability in disease presentation and progression, stratified approaches based on genetic, microbiome and metabolic profiles are essential for optimising treatment strategies. By leveraging multi‐omics data, researchers can identify biomarkers that predict disease progression and response to therapy [228]. This approach will also facilitate individualised pharmacotherapy, allowing clinicians to tailor treatment regimens based on a patient's specific metabolic profile and risk factors [229]. The implementation of precision medicine in MASLD has the potential to enhance therapeutic efficacy while minimising adverse effects.

### 7.2 Gut Microbiota Modulation as a Novel Approach

Another promising area of research is the role of gut microbiota modulation in MASLD treatment. The gut‐liver axis plays a crucial role in disease progression, and targeting gut dysbiosis has emerged as a novel therapeutic strategy. Probiotics and prebiotics, which promote a healthier gut microbiome, have demonstrated benefits in modulating systemic inflammation and reducing hepatic fat accumulation [230]. Faecal microbiota transplantation is also being explored as a potential intervention to restore gut microbial balance and improve liver health [231]. Bile acid metabolism is another key pathway being studied, as alterations in bile acid composition can impact metabolic and inflammatory processes in the liver [222].

### 7.3 Emerging and Experimental Therapies

In addition to established treatments, several emerging and experimental therapies are being investigated for MASLD. Gene therapy represents an exciting avenue, with efforts to target genetic variants such as PNPLA3, which has been implicated in MASLD progression [232]. By modulating lipid metabolism pathways, gene‐based therapies could provide personalised treatment options in the future [135]. Anti‐fibrotic agents, including fibroblast growth factor analogs [233], galectin inhibitors [234] and lysyl oxidase‐like protein 2 (LOXL2) inhibitors [235], are also under investigation for their potential to halt or even reverse fibrosis progression in MASLD and MASH patients. These novel therapies could offer targeted approaches to managing the disease, especially in patients with advanced fibrosis or cirrhosis.

## 8 Conclusion

MASLD is a multifactorial metabolic disease with systemic implications, affecting not only the liver but also increasing the risk of cardiovascular disease, chronic kidney disease and metabolic dysfunction. Its complexity necessitates a comprehensive approach to management, incorporating lifestyle modifications, pharmacological interventions and emerging therapies tailored to individual patient needs. As research continues to uncover the underlying mechanisms driving disease progression, a greater emphasis must be placed on early diagnosis, risk stratification and innovative treatment modalities.

Given the interconnection between metabolic disorders, an interdisciplinary approach is crucial for effectively managing MASLD. Collaboration between hepatologists, endocrinologists, cardiologists and experts in lifestyle medicine will enable a more holistic approach to patient care. Integrating these specialties will facilitate early intervention, optimise treatment strategies and address comorbidities that contribute to disease progression.

Future research should focus on exploring novel drug targets, developing digital health interventions and enhancing early screening methods. Advances in artificial intelligence, multi‐omics technologies and precision medicine hold the potential to revolutionise MASLD diagnosis and treatment. Furthermore, efforts to improve public health initiatives and ensure equitable healthcare access will be instrumental in reducing the overall burden of MASLD. By fostering interdisciplinary collaboration and prioritising innovative research, the medical community can make significant strides in improving outcomes for individuals affected by this increasingly prevalent disease.

## Author Contributions

D.M.M.: writing – original draft, editing, figure creation. K.F.M.: writing – original draft, editing, figure creation. K.J.D.‐S.: writing – original draft, review and editing, writing – final draft. All authors approved of the final submission.

## Conflicts of Interest

The authors declare no conflicts of interest.

## Data Availability

The authors have nothing to report.
